# A low incidence of perineal hernia when using a biological mesh after extralevator abdominoperineal excision with or without pelvic exenteration or distal sacral resection in locally advanced rectal cancer patients

**DOI:** 10.1007/s10151-020-02248-z

**Published:** 2020-06-08

**Authors:** E. A. Dijkstra, N. L. E. Kahmann, P. H. J. Hemmer, K. Havenga, B. van Etten

**Affiliations:** 1 Department of Medical Oncology, University of Groningen, University Medical Center Groningen, Groningen, The Netherlands; 2 Department of Surgery, University of Groningen, University Medical Center Groningen, Hanzeplein 1, P.O. Box 30.001, 9700 RB Groningen, The Netherlands

**Keywords:** Biological mesh, Permacol, Perineal hernia, Wound healing, Rectal cancer surgery, Extralevator abdomino perineal excision, Pelvic exenteration

## Abstract

**Background:**

Extralevator abdominoperineal excision (ELAPE), abdominoperineal excision (APE) or pelvic exenteration (PE) with or without sacral resection (SR) for locally advanced rectal cancer leaves a significant defect in the pelvic floor. At first, this defect was closed primarily. To prevent perineal hernias, the use of a biological mesh to restore the pelvic floor has been increasing. The aim of this study, was to evaluate the outcome of the use of a biological mesh after ELAPE, APE or PE with/without SR.

**Methods:**

A retrospective study was conducted on patients who had ELAPE, APE or PE with/without SR with a biological mesh (Permacol™) for pelvic reconstruction in rectal cancer in our center between January 2012 and April 2015. The endpoints were the incidence of perineal herniation and wound healing complications.

**Results:**

Data of 35 consecutive patients [22 men, 13 women; mean age 62 years (range 31–77 years)] were reviewed. Median follow-up was 24 months (range 0.4–64 months). Perineal hernia was reported in 3 patients (8.6%), and was asymptomatic in 2 of them. The perineal wound healed within 3 months in 37.1% (*n* = 13), within 6 months in 51.4% (*n* = 18) and within 1 year in 62.9% (*n* = 22). In 17.1% (*n* = 6), the wound healed after 1 year. It was not possible to confirm perineal wound healing in the remaining 7 patients (20.0%) due to death or loss to follow-up. Wound dehiscence was reported in 18 patients (51.4%), 9 of whom needed vacuum-assisted closure therapy, surgical closure or a flap reconstruction.

**Conclusions:**

Closure of the perineal wound after (EL)APE with a biological mesh is associated with a low incidence of perineal hernia. Wound healing complications in this high-risk group of patients are comparable to those reported in the literature.

## Introduction

Abdominoperineal excision (APE) is widely used in the treatment of rectal cancer when the tumour is less than 6 cm from the anal verge. The conventional technique of APE is an abdominal mobilization of the mesorectum to the distal rectum/anus followed by perineal excision close to the anal sphincter. During an extralevator APE (ELAPE), the levator ani muscle is resected en bloc with the rectum [1]. Because of this, the defect in the pelvic floor after ELAPE is larger than after conventional APE. There is a comparable perineal wound morbidity between ELAPE and conventional APE though [2, 3]. In ventrally advanced rectal tumours pelvic exenteration (PE) or resection of the posterior vaginal wall, and in dorsally advanced tumours, distal sacral resection (SR) may be considered in combination with (EL)APE. In these types of resection, the defect in the pelvic floor is more prominent, and filling of the pelvis is mainly by omentoplasty or small bowel. This significant defect in the pelvic floor may result in lower rates of wound healing.

Primary closure of the perineal wound is associated with a high rate of wound complications, especially in patients treated with neoadjuvant radiotherapy (40–45%) [4–7]. Other options for closure are a myocutaneous flap reconstruction, a lotus petal flap reconstruction or a biological mesh. A myocutaneous flap reconstruction and biological mesh reconstruction are associated with equal morbidity [8]. However, gluteal flap reconstructions result in a higher perineal hernia rate than those with biological mesh (21% vs 0, *p* < 0.01) [9]. After a lotus petal flap reconstruction, Clavien-Dindo grade I-II complications occurred in 46% of patients [10]. The use of a biological mesh is promising since the perineal herniation rate has been lowered to 0–13% [4, 9, 11–14]. Besides, a systematic review showed improved pelvic wound healing, shortened operation time and earlier postoperative mobilization after biological mesh compared to primary closure [15].

In the University Medical Center Groningen (UMCG), we have used biological mesh reconstructions after ELAPE, APE with or without PE or SR between January 2012 and April 2015. In this paper, we describe our results with an emphasis on perineal hernia and wound healing.

## Materials and methods

A total of consecutive 35 patients who had surgery for rectal cancer with or without PE or SR with the reconstruction of the pelvic floor using a biological mesh (Permacol™; Covidien, Mansfield, MA, USA), at the UMCG between January 2012 and April 2015, were evaluated. Patient, preoperative, surgery and complication and toxicity characteristics were collected.

All patients had pelvic floor reconstruction with a Permacol™ Surgical implant. This Permacol™ mesh is a porcine dermal collagen implant from which cells, ribonucleic acid and deoxyribonucleic acid are removed. The resulting acellular collagen matrix is then cross-linked to enhance durability. The size of the Permacol™ mesh depended on the size of the defect of the pelvic floor. The mesh was fixated with prolene sutures to the pelvic sidewall and the remnants of the levator muscle, the presacral fascia and the remnants of the puborectal sling. This part of the operation was performed in a prone jackknife or knee-chest position. All patients had reconstruction by omentoplasty. First, adhesions of the small bowel, colon or abdominal omentum were released. Then the omentum was cleared from the transverse colon and the stomach. The omentoplasty was pedicled on the right gastroepiploic artery and placed in the small pelvis. However, in some cases, the defect area was also covered with a de-epithelialized lotus petal flap. The flap was de-epithelialized so the extensive soft tissue could be used to fill dead spaces to prevent fluid accumulation causing complications. The lotus petal flap reconstruction technique is a perforator flap which can be mono- or bilateral and has been described earlier by our group [10]. In short, in the gluteal or inguinal fold the length and width of the flap are marked preoperatively. After resection, the skin of the flap is de-epithelialized and incised until the underlying fascia of the muscle is mobilized by elevating the flap from lateral to medial. The skin is closed in layers and finished with sutures.

Complications were graded using the Clavien-Dindo classification [15]. We did not differentiate between early and late complications. The treatment of wound complications was divided into five groups with conservative treatment, antibiotics, vacuum-assisted closure (VAC), surgical re-intervention or a secondary lotus petal flap reconstruction as possible treatment options. The following United States Food and Drug Administration (FDA) definition of complete wound healing was used: 100% re-epithelialization of the wound surface with no discernable exudate and without drainage or dressing requirement, confirmed at outpatient clinic visits [16]. A computed tomography (CT) scan was used in the follow-up of these rectal cancer patients and also to detect perineal hernia.

The association between wound dehiscence and multiple variables (sex, body mass index (BMI), smoking, diabetes mellitus, corticosteroids, cancer stage, American Society of Anesthesiologists (ASA) classification, neoadjuvant therapy, type of surgery, lotus petal flap reconstruction, intraoperative brachytherapy (IOBT), wound closure, perineal hernia, decease and cause of death) were calculated using the Pearson’s Chi-square test. A *p*-value of < 0.05 was considered significant. All analyses were performed using SPSS, version 23.0 (SPSS, Chicago, IL, USA).

## Results

### Patient characteristics

Table 1 shows the baseline characteristics. The median length of follow-up was 24 months (range 0.4–64 months). Almost all patients had resection for a primary or recurrent rectal adenocarcinoma; one patient was treated for a rectal gastrointestinal stromal tumour (GIST). All of the adenocarcinoma patients had neoadjuvant radiotherapy (Table 2).

**Table 1:** Baseline characteristics

	Total patient group	Wound dehiscence	*p* value
	(*n* = 35)	(*n* = 18)	
Sex			0.90
Male	22 (62.9)	11 (61.1)	
Female	13 (37.1)	7 (38.9)	
Age at randomization in years*	62 (31–77)	60 (31–75)	
BMI kg/m^2^**	28 (20–38)	27 (18–38)	
Smoking			0.56
Yes	9 (25.7)	6 (33.3)	
No	26 (74.3)	12 (66.7)	
Diabetes mellitus			0.97
Yes	4 (11.4)	2 (11.1)	
No	31 (88.6)	16 (88.9)	
Corticosteroids			0.34
Yes	5 (14.3)	1 (5.6)	
No	30 (85.7)	17 (94.4)	
Indication			0.61
GIST	1 (2.9)	0 (0.0)	
Adenocarcinoma	33 (97.1)	18 (100.0)	
T3	8 (22.9)	4 (22.2)	
T4	16 (45.7)	6 (33.3)	
Recurrence	10 (28.6)	8 (44.4)	
ASA physical status			0.48
1	8 (23.5)	3 (16.7)	
2	24 (70.6)	15 (83.3)	
3	2 (5.7)	0 (0.0)	

*BMI* body mass index, *GIST* gastrointestinal stromal tumour, *ASA* American Society of Anesthesiologists

Data are presented as *N* (%). *mean (range), **median(range)

**Table 2:** Treatment characteristics

	Total patient group	Wound dehiscence (*n* = 18)	*p* value
	(*n* = 35)		
Neoadjuvant radiotherapy			0.67
50.0/50.4 Gy	20 (57.1)	9 (61.1)	
30.0/30.6 Gy	7 (20.0)	6 (33.3)	
5 × 5 Gy	7 (20.0)	3 (16.7)	
No radiotherapy (GIST tumor)	1 (2.9)	0 (0.0)	
Surgery			0.56
ELAPE without distal sacrum resection	8 (22.9)	3 (16.7)	
ELAPE with distal sacrum resection	5 (14.3)	1 (5.6)	
APE without distal sacrum resection	5 (14.3)	4 (22.2)	
APE with distal sacrum resection	10 (28.6)	8 (44.4)	
Pelvic exenteration	7 (20.0)	2 (11.1)	
Lotus petal flap			0.52
Yes	7 (20.0)	5 (27.8)	
No	28 (80.0)	13 (72.2)	
IOBT			0.38
Yes	3 (8.6)	3 (16.7)	
No	32 (91.4)	15 (83.3)	

Data are presented as *N* (%)

*ELAPE* extralevator abdominoperineal resection, *APE* abdominoperineal resection, *GIST* gastrointestinal stromal tumour, *IOBT* intraoperative brachytherapy

### Treatment characteristics

In total, 35 consecutive patients had surgery for rectal cancer and a Permacol™ mesh was used. Nine patients (25.7%) had previous surgery for rectal cancer. In total, an ELAPE was performed in 13 patients (37.1%) and an APE in 15 patients (42.9%). Excision of the distal sacrum was performed in 61.5% of the ELAPE patients and in 66.7% of the APE patients. The other patients (20.0%) underwent PE, which includes excision of the uterus, vagina and/or bladder in female, or bladder and prostate in male patients (Table 2).

All patients had reconstruction by omentoplasty. In 7 patients (20.0%), a lotus petal flap reconstruction was performed immediately following the rectal resection. In 2 other patients, a lotus petal flap reconstruction was performed secondarily, because of invalidating wound healing problems after initial primary skin closure.

Three patients (8.6%) received intraoperative brachitherapy (IOBT) with a dose of 10 Gy during surgery, because of a peroperatively suspected close resection margin. In all 3 patients, there was postoperative wound dehiscence.

### Wound dehiscence

Eighteen patients (51.4%) had minor to major wound dehiscence, for which treatment was conservative in 8 cases (grade I complication). One patient received antibiotic treatment (grade II complication). A wound dehiscence grade III complication occurred in 9 patients (27%): 5 patients needed VAC therapy, and the other 4 patients required a reoperation with the use of lotus petal flap in 2 cases (Table 3).

**Table 3:** Wound characteristics

	Total patient group	Wound dehiscence	*p* value
	(*n* = 35)	(*n* = 18)	
Wound healing			0.79
0–30 days	2 (5.7)	0 (0.0)	
30–90 days	11 (31.4)	3 (16.7)	
90–180 days	5 (14.3)	3 (16.7)	
180–365 days	4 (11.4)	3 (16.7)	
> 365 days	6 (17.1)	4 (22.2)	
Deceased*	4 (11.4)	2 (11.1)	
Lost to follow-up	3 (8.6)	3 (16.7)	
Wound dehiscence			
Clavien-Dindo I		8 (23.5)	
Clavien-Dindo II		1 (5.6)	
Clavien-Dindo III		9 (50.0)	

Data are presented as *N* (%)

*Unclear whether the wound was healed before death

None of the variables sex, BMI, smoking, diabetes mellitus, corticosteroids, cancer stage, ASA classification, neoadjuvant therapy, type of surgery, lotus petal flap reconstruction, IOBT wound closure, perineal hernia, decease and cause of death were associated with an increased risk of wound dehiscence (Tables 1, 2, 3, 4).

**Table 4:** Outcome

	Total patient group (*n* = 35)	Wound dehiscence (*n* = 18)	*p* value
Perineal hernia			0.20
Cases	3 (8.6)	0 (0.0)	
Asymptomatic	2 (66.6)	0 (0.0)	
Symptomatic	1 (33.3)	0 (0.0)	
Deceased			0.50
Cases	18 (51.4)	11 (61.1)	
Months after surgery*	24 (0.3–67)	25 (2–47)	
Cause of death			0.83
Tumour recurrence and/or metastases	14 (77.8)	8 (72.7)	
Sepsis and MOF	1 (5.6)	1 (9.1)	
Pulmonary embolism	1 (5.6)	0 (0.0)	
Unknown	2 (11.1)	2 (18.2)	

*MOF* multi-organ failure

Data are presented as *N* (%) *mean(range)

### Wound healing

Table 3 shows wound healing (according to the FDA definition of a completely healed wound [16]) and wound complications following surgery with Permacol™ mesh closure. The wound was healed within 30 days after surgery in 2 patients (5.7%) and within 3 months after surgery in 11 patients (31.4%). In total, the wound was completely healed within 1 year after surgery in 22 patients (62.9%). As regards the remaining 13 patients (37.1%), the wound was completely healed after 1 year in 6 patients (17.1%), 3 patients (8.6%) were lost to follow-up and 4 patients (11.4%) died before confirmation of wound healing during outpatient clinical visits (Table 3).

One patient experienced a deep wound infection 3 weeks after the initial surgery. The Permacol™ mesh lay loose in the pelvis and it was removed.

### Perineal hernia

A perineal hernia occurred in 3 patients (8.6%). This happened 10, 13 and 23 months after surgery. None of them experienced a wound infection after surgery and before the occurrence of the perineal hernia. One of the patients with perineal hernia smoked and had diabetes mellitus, another patient smoked, and the third patient used corticosteroids. Two of the hernias were asymptomatic, so no further treatment was necessary. The other patient underwent surgical correction of the hernia (Table 4). Two of the patients with an asymptomatic perineal hernia had an ELAPE, the other patient ( with a symptomatic perineal hernia) an APE. Of the 7 patients who were lost to follow-up or died before the wound was healed, only 1 patient (14.3%) developed a perineal hernia.

### Mortality

In total, 18 patients (51.4%) died, one of them within 30 days after surgery, due to a pulmonary embolism on day 11. Another patient died as a result of sepsis and multi-organ failure within 3 months after surgery. In 11 patients progression and/or recurrence of disease was the cause of death during follow-up. The cause of death was unknown in 3 patients (Table 4).

## Discussion

In this study, we demonstrated a low rate of perineal hernia after the use of a biological mesh in rectal cancer patients. Moreover half of the perineal wounds closed within 6 months.

The perineal hernia rate when a biological mesh was used was 8.6%. Only 2 patients (5.7%) experienced a perineal hernia after ELAPE, which were both asymptomatic. The currently most extensive series of biological mesh repair after ELAPE, reported by Thomas et al., demonstrated a comparable asymptomatic perineal hernia rate of 7% [17]. When biological mesh was combined with a lotus petal flap, the incidence of perineal hernia was low (*n* = 1) in our study. This perineal hernia rate is comparable to that in the study of Hellinga et al. in which none of the patients experienced a perineal hernia after the combination of a lotus petal flap with a biological mesh [10]. In the multicenter BIOPEX-study, 101 patients were randomized between primary closure of the perineum or closure with a biological mesh (non-crosslinked Strattice™ Reconstructive Tissue Matrix from LifeCell, Allergan^©^) [13]. After 1-year of follow-up, the perineal hernia rate was significantly lower in patients with a biological mesh (13% after biological mesh, 27% after primary closure, *p* = 0.03) [13]. Besides, among the 228 patients in a retrospective study, significantly less perineal hernias developed after use of a biological mesh instead of primary closure (3.4% vs 13.0%, *p* = 0.02) [11]. In the study of Sayers et al., 56 pateints underwent ELAPE had primary closure with (*n* = 8) or without (*n* = 38) biologic mesh or flap [18]. A total of 14 patients (25.9%) developed a perineal hernia including both patients who received a biological mesh reconstruction, and 12 of the 38 patients who were closed with sutures [18]. The retrospective study of Christensen et al. compared the use of a gluteal flap with a biological mesh in 57 patients [9]. A perineal hernia developed in 7 patients with a gluteal flap reconstruction vs. none of the patients in the mesh group (*p* < 0.01) [9]. It seems that the chance of developing a perineal hernia is smaller after using a biological mesh than with primary closure and gluteal flap. However, data is limited. The clinical review by Foster et al. stated that the decision regarding which method should be performed should depend on surgical expertise, morbidity, cost-effectiveness and the size of the defect [7].

More than half of the patients in our study experienced minor to major wound healing problems. Half of them (*n* = 9) needed invasive treatment for these problems. One patient experienced a deep wound infection 3 weeks after the initial surgery. The mesh lay loose in the pelvis and it has been removed. Just like in our study, all patients in the BIOPEX-study received preoperative radiotherapy [13]. The BIOPEX-study showed no significant difference in perineal wound complications 30 days after surgery between the primary closure (66%) and biological mesh (63%) groups (*p* = 0.72) [13]. In the prospective study of Peacock et al., all 34 patients received a biological mesh, and 82,4% received radiotherapy up to 45 Gy [12]. This study reports an overall rate of perineal wound complications of 32%, 8%of which needed invasive treatment [12]. Grade III wound complication occurred in 7 patients (7%) in the study by Thomas et al 4 of whom needed VAC therapy [17]. In total, 71% of patients in this observational study were preoperatively irradiated with 45 Gy [17]. Christensen et al. reported a higher perineal wound infection rate after the use of biological mesh (17%) compared to a gluteal flap reconstruction (6%) although this difference was found not significant (*p* = 0.26) [9]. However, Christensen et al. described wound infection as an infection requiring surgical intervention by operative irrigation and/or debridement or VAC [9]. If we use the same criteria, we find a wound infection rate of 14%. In total, 44% of patients in the study of Sayers et al., experienced perineal complications. Twenty-two of them (39.3%) received preoperative radiotherapy [18]. On the other hand, none of the patients (*n* = 17) in the study by Ge et al., developed perineal complications after the use of a biological mesh [19]. However, the patients in this study did not receive preoperative radiotherapy [19]. In preoperatively irradiated patients who had APE with primary closure, wound complications occur in 40–45% [4–6].

In an ongoing multicenter trial, the NEAPE-trial, 487 patients will be randomized (1:1) between the use of a porcine-collagen implant or the use of a gluteus maximus muscle. The aim is to investigate whether a porcine-collagen implant is superior or equally beneficial to a gluteus maximus reconstruction. We are awaiting the results.

Wound healing problems in this specific surgery, are most likely caused by multiple factors. The most critical risk factor, which also emerges from the literature above, is preoperative radiotherapy [4–6, 11, 20]. A long operative time, more extensive surgery, contamination of the operating field, conventional primary closure, intraoperative bowel-perforation and the location of the wound are other possible risk factors [6, 11, 21]. With the use of a biological mesh, an anatomical dead space is created between the mesh and perineal skin, in which exudate can lead to abscess formation.

Limitations of our study include the small study population and the retrospective nature of the study. Another limitation is that the use of the biological mesh was not compared with other methods of wound closure. There was no systematic follow-up interval regarding wound inspection and no scale for wound observation was used.

## Conclusions

Our study demonstrates that the use of a biological mesh is a safe and feasible method for pelvic reconstruction and wound closure after ELAPE, APE or PE with or without SR in patients with locally advanced or recurrent rectal cancer and multiple wound healing risk factors. Moreover, the use of biological mesh is associated with a low rate of perineal hernia.
